# Combined or Sequential Treatment with Immune Checkpoint Inhibitors and Car-T Cell Therapies for the Management of Haematological Malignancies: A Systematic Review

**DOI:** 10.3390/ijms241914780

**Published:** 2023-09-30

**Authors:** María Antonia Pérez-Moreno, Pablo Ciudad-Gutiérrez, Didiana Jaramillo-Ruiz, Juan Luis Reguera-Ortega, Laila Abdel-kader Martín, Sandra Flores-Moreno

**Affiliations:** 1 Department of Pharmacy, University Hospital Virgen del Rocío, 41013 Seville, Spain; 2 Department of Haematology, University Hospital Virgen del Rocío, Instituto de Biomedicina de Sevilla (IBIS)/CSIC, University of Seville, 41012 Seville, Spain; 3 Department of Pharmacy and Pharmaceutical Technology, University of Seville, 41012 Seville, Spain

**Keywords:** checkpoint inhibitors, chimeric antigen receptor-T, combined therapy, haematologic tumour, systematic review

## Abstract

The aim of this paper was to review the available evidence on the efficacy and safety of combined or sequential use of PD-1/PD-L1 immune checkpoint inhibitors (ICI) and CAR-T cell therapies in relapsed/refractory (R/R) haematological malignancies. A systematic literature review was performed until 21 November 2022. Inclusion criteria: cohort studies/clinical trials aimed at evaluating the efficacy and/or safety of the combination of CAR-T cell therapy with PD-1/PD-L1 inhibitors in R/R haematological malignancies, which had reported results. Those focusing only on ICI or CAR-T separately or evaluating the combination in other non-hematological solid tumours were excluded. We used a specific checklist for quality assessment of the studies, and then we extracted data on efficacy or efficiency and safety. A total of 1867 articles were identified, and 9 articles were finally included (early phase studies, with small samples of patients and acceptable quality). The main pathologies were B-cell acute lymphoblastic leukaemia (B-ALL) and B-cell non-Hodgkin’s lymphoma (B-NHL). The most studied combination was tisagenlecleucel with pembrolizumab. In terms of efficacy, there is great variability: the combination could be a promising option in B-ALL, with modest data, and in B-NHL, although hopeful responses were received, the combination does not appear better than CAR-T cell monotherapy. The safety profile could be considered comparable to that described for CAR-T cell monotherapy.

## 1. Introduction

CAR-T cell therapies (chimeric antigen receptor-T cell) are a type of adoptive cell transfer therapy classified as an advanced therapy medicinal product. It is an innovative immunotherapy that involves a high social and health impact and an economic one. It consists of T-lymphocytes, which are genetically modified ex vivo to insert a gene in order for the lymphocytes to express on their surface CAR receptors, which will specifically bind to cells that express their target (including tumour cells) to destroy them [1].

Currently, several CAR-T cell-based medicines have received approval from the European Medicines Agency (EMA): tisagenlecleucel (Kymriah^®^), indicated refractory B-cell acute lymphoblastic leukaemia (B-ALL) and relapsed or refractory (R/R) diffuse large B-cell lymphoma (DLBCL) or follicular lymphoma (FL), axicabtagene ciloleucel (Yescarta^®^), authorised for the treatment of adult patients with DLBCL, primary mediastinal large cell B-cell lymphoma (PMBCL) and FL, lisocabtagene maraleucel (Breyanzi^®^) for DLBCL, PMBCL, and FL, brexucabtagene autoleucel (Tecartus^®^), for patients with R/R mantle cell lymphoma or ciltacabtagene autoleucel (Carvykti^®^) and idecabtagene vicleucel (Abecma^®^) for refractory multiple myeloma. In addition, some non-industrially manufactured CAR-T cell therapies have achieved PRIME (priority medicines) designation and EMA approval, such as ARI-0001 for refractory ALL [2,3,4,5,6,7,8,9,10].

These therapies have demonstrated good response rates and an impact on disease-free survival in patients with haematological malignancies with a very poor prognosis who have failed various lines of treatment [11,12,13,14]. Thus, they are considered to be a revolutionary strategy not only for managing haematology pathologies but also for multiple solid tumours and even non-nonhematologic diseases. However, these therapies are not exempt from limitations.

On the one hand, they have been associated with the development of unexpected serious, potentially life-threatening toxicities, including cytokine release syndrome (CRS), neurotoxicity, and “on-target/off-tumour” target recognition [2,3,4,15].

On the other hand, although the efficacy results shown in clinical trials are promising, we are in an area of great uncertainty [16]. Since the clinical trial results may not be extrapolated to patients receiving the therapy in real life, as reflected in the preliminary data published in our setting [7].

Therefore, the main challenges in developing and using CAR-T cell therapies focus on reducing the associated high toxicity and prolonging disease-free survival and overall survival. Possible solutions could be determining predictors of relapse after CAR-T-cell therapy and designing synergistic mechanisms to enhance, promote, or prolong responses to the therapy. In this regard, the combination of CAR-T cells with immune checkpoint molecule inhibitors (ICIs), such as programmed cell death-1 (PD-1) or programmed death ligand-1 (PD-L1) inhibitors, has been postulated as a hopeful strategy [17,18,19]. The basis of this strategy is that the inhibitory signals CAR-T cells encounter in the tumours microenvironment often affect cell expansion and their efficacy. PD-1 proteins in T-cells, when combined with PD-L1 in tumour cells, can induce dysfunction and depletion of the modified T-cells and contribute to an insufficient CAR-T cells efficacy [20]. Consequently, some studies have assessed immune checkpoints after CAR-T cell therapy, detecting increased surface levels of PD-1 on anti-CD19 CD4+ and CD8+ CAR-T cells after treatment and higher PD-1 expression in CAR-T cells than in non-CAR-T cells. Furthermore, genetic analyses of patients from the ZUMA-1 trial also showed increased gene expression of PD-1, LAG-3, and CTLA-4 after treatment with CD19-specific CAR-T cells [21].

With these data, some initial clinical cases combined the PD-L1 antibody with CAR-T cells to overcome tumour immune evasion from CAR-T cells reported an increased anti-tumour effect.

In 2017, Chong et al. [22] published the case of a patient with progressing DLBCL who was first treated with anti-CD19 CAR-modified T-cells and subsequently with an anti-PD-1 antibody. After administration of the two therapies, the patient had a clinically significant anti-tumour response, an increase in CAR-T cell expansion, and a decrease in PD-1 co-expression in CAR-T cells. More recently, Hill et al. [23] treated with nivolumab at 3 mg/kg on day 11 of a patient with LBDCG progressing after axi-cel therapy, which showed rapid responses to treatment and a dramatic increase in the number of CAR-T cells after a PD-1 blockade [15]. Niu et al. [24] presented a 61-year-old male with refractory DLBCL who received CAR-T cell therapy and nivolumab at 3 mg/kg prior to lymphodepletion. The expression of PD-1 in T-cells was increased (52.7%) with a significant therapeutic effect, a large expansion of the CAR-T cells, and the patient remained disease-free more than 12 months later.

In addition, many clinical trials suggest that the use of checkpoint inhibitors may be an effective and safe strategy in optimising CAR-T cell therapy and may improve the efficacy and persistence of CAR-T cells in patients with B-ALL, DLBCL, B-cell non-Hodgkin’s lymphoma (B-NHL), FL, etc. [25]. However, CAR-T cell combination therapy with an inhibitory checkpoint PD-1 blockade could exhibit potential side effects, especially cytokine release syndrome (CRS). Nevertheless, new studies examining the combined efficacy of both therapeutic options are in progress.

Based on the above, this study aims to conduct a literature review to analyse the available evidence on the efficacy and safety of the combined or sequential use of PD-1 or PD-L1 receptor inhibitors and CAR-T cell therapies for the management of haematological malignancies in relapse or refractory to other treatments.

## 2. Material and Methods

### 2.1. Literature Search

**Figure 1:**
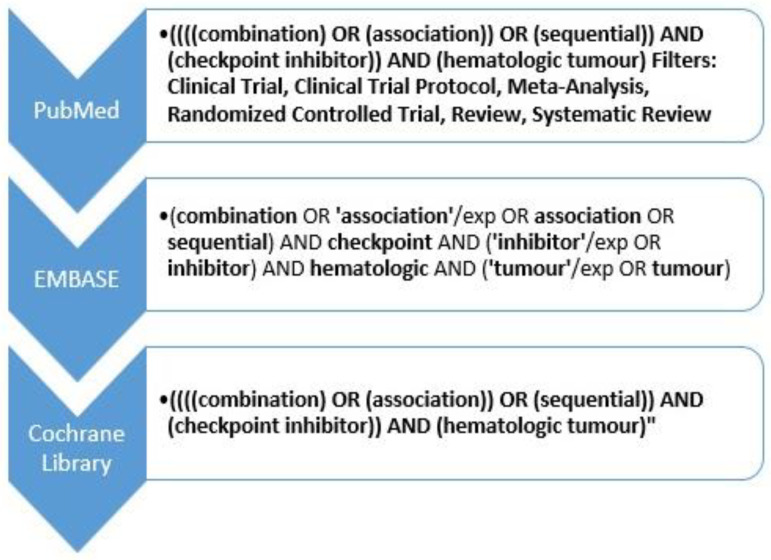
Search strategy.

A comprehensive literature review with no language or publication date restrictions was conducted in three main biomedical databases (MEDLINE, EMBASE, and the Cochrane Library) up to November 2022. The search strategy is detailed in Figure 1.

The search was completed manually by consulting websites of drug regulatory agencies, oncology–haematology scientific societies (and other bodies that might contain information related to the subject) to identify any other relevant reports that provided more information about CAR-T cell combination therapy with an inhibitory checkpoint PD-1 blockade. A cross-reference search of the literature was also performed using the references in the localised literature.

We reviewed the Preferred Reporting Items for Systematic Reviews and Meta-Analyses (PRISMA) guidelines [26], and the review was registered in the International Prospective Register of Systematic Reviews (PROSPERO) Database International Prospective Register of Systematic Reviews with ID CRD42023406375.

### 2.2. Eligibility Criteria

#### 2.2.1. Inclusion Criteria

We included studies or clinical studies (published or active with preliminary published results) aimed at evaluating the efficacy and/or safety of the simultaneous or sequential combination of CAR-T cell therapy with PD-1 or PD-L1 inhibitors in patients with haematological malignancies in relapse or refractory to other treatments. The most up-to-date data analysis was selected in those publications that refer to the same study but present different cut-off points for outcome assessment. In terms of design, clinical trials and cohort studies were included (given the lack of evidence).

#### 2.2.2. Exclusion Criteria

We excluded studies or clinical trials focusing solely on immune checkpoint therapy (without association to CAR-T cell therapy) or studies evaluating the combination in other non-haematological solid tumour types and those studies/trials in progress for which no results were available.

In addition, publications reporting a single clinical case, including any other study design, were excluded.

### 2.3. Study Selection

In the first phase, authors deleted duplicates after the literature search in databases. Articles were selected by reviewing the title and abstract and accessing the full text of the article to extract data results. To minimize the risk of bias, two reviewers (M.A.P.M. and P.C.G.) performed this procedure independently; a third party resolved their disagreements. Subsequently, the authors carried out a critical reading of said selected full-text articles.

### 2.4. Data Extraction and Quality Assessment

We designed an ad hoc table where information about the main characteristics of the selected studies was collected, apart from additional tables that gathered the main efficacy and safety results of each study. Moreover, the authors performed a qualitative synthesis of the data results, in which patients with acute leukaemia and lymphoma pathologies were analysed separately. The degree of heterogeneity of the data did not allow a meta-analysis of the results.

Two authors (M.A.P.-M. and P.C.-G.) independently assessed the quality of each report with a specific checklist of the Scottish Intercollegiate Guidelines Network guidelines (Methodology checklist 3: cohort studies) [27].

### 2.5. Study Endpoints

The efficacy variables included were overall survival (OS), progression-free survival (PFS), response to treatment defined as complete response (CR), partial response (PR), stable disease (SD), or disease progression (DP) including overall response rate (ORR) and existence of CAR re-expansion after ICI administration if applicable. In addition, the main toxicities associated with the investigational drugs were reviewed.

The statistical analysis was carried out using Excel 2007 and IBM SPSS Statistics software, 19th version. Qualitative variables were shown as percentages, and quantitative variables as central measures (mean) with dispersion measures (standard deviation and range).

## 3. Results

### 3.1. Literature Search Results

**Figure 2:**
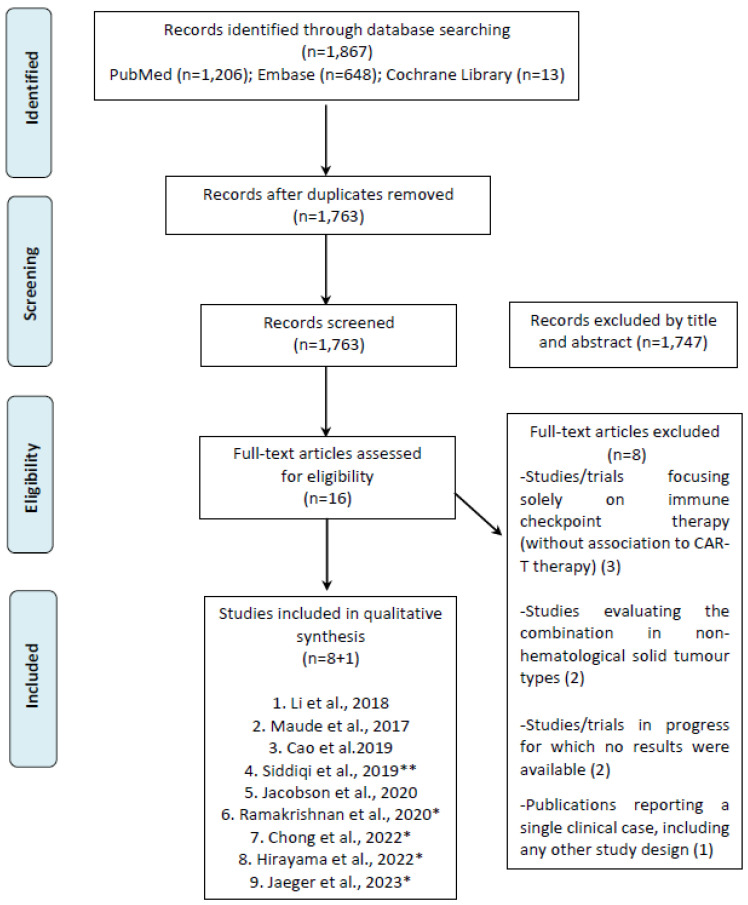
Study selection flowchart. * These studies were also hand-searched to identify any other relevant studies that evaluated and provided more information about efficacy or safety. ** This study was found in the grey literature [28,29,30,31,33,34,35,36,37].

A total of 1867 articles were identified (1206 in MEDLINE, 648 in EMBASE, and 13 in the Cochrane Library). After removing duplicates, 1763 were reviewed by title/abstract. Finally, nine articles met the established selection criteria: two articles focused on B-ALL and seven studies on B-NHL. Figure 2 shows the flowchart of selecting documents during the systematic review.

The main reasons for exclusion were the study of other oncological pathologies (not haematological) and the object of analysis different from that established, fundamentally the study of the effectiveness of ICI and CAR-T therapy separately and not in association (simultaneous or sequential). Some of them were clinical cases that referred to a single patient and laid the foundations for implementing larger-scale studies or CE included in this work.

### 3.2. Clinical Study Features and Efficacy Results

The main features and characteristics of the studies are detailed in Table 1.

An overall assessment of the internal validity of the nine selected studies was carried out. These studies met most of the criteria, so they were scored as “acceptable” by the authors. However, potential confounders were not considered in the design and analysis of each report, and confidence intervals were not often mentioned in the statistical analysis.

Table 2 summarizes efficacy data more recently reported by the selected articles. A brief description of all reports is provided below:

### 3.3. LLA-B

Li et al. [28] performed a study at the Children’s Hospital of Philadelphia to investigate the efficacy of combining PD-1 inhibitors with anti-CD19 CART Therapy in paediatric patients with R/R multi-line B-ALL and lymphoblastic lymphoma. The patients included in the study had demonstrated early loss of CAR or partial response (PR) or had no CAR-T cells present. The treatment regimen involved administering the PD-1 inhibitor at least 14 days after CAR infusion and after resolving LCH (cytokine release syndrome) symptoms. Additionally, patients had the option of receiving repeated doses of the PD-1 inhibitor, up to every 3 weeks. Three out of six of patients treated with CAR-T in combination with the PD-1 inhibitor for early B-cell recovery restored B-cell aplasia over an interval ranging from 5 to 15 months, two of whom had persistent aplasia during pembrolizumab treatment. Four patients were treated with pembrolizumab for bulky extramedullary disease that did not respond or relapsed after CAR-T therapy, resulting in two PRs and two CRs. In the other four patients who did not achieve disease remission with CAR-T, no CR was obtained with the addition of pembrolizumab, although PRs were observed, and one patient progressed with CD19-negative disease.

Maude et al. [29] conducted a clinical trial in LLA-B patients with PR/null or a history of anti-CD19 CAR-T cell deficient persistence occurring 14 days-2 months post-infusion. They received 1–3 doses of pembrolizumab. Results about four patients have been published in ASCO. Pembrolizumab prolonged the persistence of circulating CAR-T cells in all four children, with objective responses in two of them. One of the responders received reinfusion of CAR-T combined with pembrolizumab after a CD19-positive relapse to CAR-T therapy with poor CAR-T cell persistence; CR with prolonged CAR-T persistence was achieved. The other patient received a PD-1 inhibitor because of widespread extramedullary involvement at 1 month post infusion despite having remission of the disease in bone marrow, with a large increase in CAR expansion and a significant reduction of disease on a 3-month PET-CT scan.

### 3.4. B-NHL

Cao et al. [30] conducted a cohort study of 11 patients diagnosed with R/R lymphoma who were treated with anti-CD19 CAR-T therapy and nivolumab 3 mg/kg as a single dose on day +3 after CAR-T cell infusion. Response to therapy was assessed using PET-CT and bone marrow at 6 weeks post-infusion and analysed for CD19 CAR-T cell expansion, PD-1 expression level, lymphocyte subpopulations, and cytokine levels. Nine patients achieved an objective response, and with a median follow-up of 6 months (1–15), the median PFS was 6 months, and three patients remained in response at the time of analysis. The two NR patients deteriorated rapidly. The expression of PD-1 on T cells was significantly decreased after nivolumab, but they did not find an association between PD-1 levels and response to the treatment.

In 2019, Siddiqi et al. [31] reported the preliminary data of their phase 1/2 PLATFORM study (within the TRANSCEND NHL 001 trial, NCT02631044) [32], evaluated liso-cel in combination with durvalumab (anti-PD-L1 antibody) in patients with R/R B-NHL. At data cut-off, 11 pts (dose level-1 n = 8; dose level-2 n = 3) completed at least one durvalumab, 10 patients responded to the treatment, and 3 of the first 6 patients treated showed increased CAR-T cells at day 85 compared with pre-durvalumab levels on day 29. One patient maintained CAR-T cells near peak expansion levels until day 85.

**Table 1:** Clinical study features and disease-specific characteristics of the patients.

Author, Year	Type of Study	Age (Eligible)	Treatment/Period/Regimen	Patients (N)	Pathology (N)	Disease Status at Treatment (N)
Li et al. [28], 2017	Single institution Pilot clinical trial	Range: 4–17 years	CD19-directed CAR-T cell therapy in combination with pembrolizumab (n = 13) or nivolumab (n = 1)	14	B-ALL (13) B-LL (1)	Relapsed (13) Refractory (1)
Maude et al. [29], 2017	Phase I/pilot trial	Range: 1–24 years	1–3 doses of pembrolizumab starting 14 d-2 months post Anti-CD19 CAR-T cell infusion	4	B-ALL (4)	Relapsed
Cao et al. [30], 2019	Cohort study	Median age: 65 years (range 26–75)	Nivolumab 3 mg/kg in a single dose on the 3rd day after CD19 CAR-T cell infusion	11	DLBCL (10) -Stage III (2) -Stage IV (8) BL/IV (1)	Primary refractory (5) Relapsed (6)
Siddiqi et al. [31], 2019 (PLATFORM study)	Multiarm, parallel cohort, phase 1/2 study	Range: 53–78 years	Liso-cel at 1 of 2 dose levels (DL): DL1 = 50 × 10e6 or DL2 = 100 × 10e6 CAR- T cells Durvalumab 1500 mg every 4 weeks as an IV infusion from day 29 at a total dose of for up to 12 months	18 (11 *)	DLBCL (10) FL (1)	Refractory Relapsed
Jacobson et al. [33], 2020 (ZUMA-6 trial)	Multi-centre open-label phase I/IIl study	≥18 years	A single infusion of KTE-C19 CAR-T cells IV followed by four doses of atezolizumab 1200 mg/dose IV every 3 weeks, beginning after 21 days (Phase 1 Cohort 1), 14 days (Phase 1 Cohort 2) or 1 day (Phase 1, Cohort 3, and Phase 2)	28	DLBCL (28)	Refractory (28)
Ramakrishnan et al. [34], 2020 (ALEXANDER trial)	Single Arm, multi-centre, open-label, phase I/II study	Median age: 59 (range 28–83)	AUTO3 alone, or with three doses of pembrolizumab 200 mg every 3 weeks starting on D14 (regimen A), or with a single dose of pembrolizumab 200 mg on D-1 (regimen B)	33 (29 *)	DLBCL-NOS (25) t-DLBCL (6) High-grade B-cell lymphoma (2)	Refractory (26)
Chong et al. [35], 2022	Prospective, open-label, single-institution, phase I/IIa study	Median age: 58 years (range 30–78)	After treatment with CART19 Pts received a fixed dose of pembrolizumab 200 mg IV every 3 weeks until the progression of disease, therapy limiting-toxicity, or elective protocol discontinuation	12	DLBCL (11) -GCB-THL (3) -T-cell rich DLBCL (1) -tFL (1) FL (1)	Refractory (9) Relapsed (3)
Hirayama et al. [36], 2022	Multi-centre, open-label phase Ib trial.	Median age: 58 years (range 32–69)	(a) Durvalumab (225 mg/750 mg/1500 mg) 21 and 28 days after JCAR014 (b) Durvalumab (7.5 mg/22.5 mg/75 mg, 225 mg/750 mg or 1500 mg) 1 day before JCAR014 infusion and every 4 weeks (until PD or unacceptable toxicity, maximum 10 doses)	29 (26 *)	B-cell NHL -DLBCL (13) -t-DLBCL (8) -High-grade B-cell lymphoma (6) -Other (2)	Refractory Relapsed
Jaeger et al. [37], 2023 (PORTIA trial)	Multi-centre, open-label, phase Ib study	≥18 years	Single tisagenlecleucel (CTL019) IV on Day 1 and Pembrolizumab 200 mg every 3 weeks, for up to six doses starting on day 15 after (in cohort 1), on day 8 or 22 (in subsequent cohorts)	15 (12 *)	DLBCL (12)	Refractory Relapsed

* Evaluable for response. Abbreviations: B-ALL, B-cell acute lymphoblastic leukaemia; BLL, B-lymphoblastic lymphoma; B cell-NHL, B-cell no Hodgkin; lymphoma DLBCL, diffuse large B-cell lymphoma; tFL, transformed follicular lymphoma; THL, triple-hit lymphoma; rBCL, rearrangement B-cell lymphoma; BL, Burkitt lymphoma; PMBCL, primary mediastinal B-cell Lymphoma.

**Table 2:** Overview of relevant efficacy results of selected studies.

Author, Year	Pathology	Study Treatment	Patients (N)	Response N (%)	Re-Expansion CAR N (%)	PFS (Months)	OS # (%)	DOR # (%)
Li et al. [28], 2017	B-ALL	Anti-CD19 + Nivolumab or Pembrolizumab	14	ORR: 6 (42.9%) - CR: 2 (14.3%) - PR: 4 (28.6%) PD: 1 (7.1%)				
Maude et al. [29], 2017	B-ALL	Tisa-cel+ Pembrolizumab	4	ORR: 2 (50.0%) - CR: 1 (25.0%) - PR: 1 (25.0%)	4 (100%)			
Cao et al. [30], 2019	B cell-NHL	Anti-CD19+ Nivolumab	11	ORR: 9 (81.8%) - CR: 5 (45.5%) - PR: 4 (36.4%)		6 (1–14)		
Siddiqi et al. [31], 2019 (PLATFORM study)	B cell-NHL	Liso-cel + Durvalumab	18 (11 *)	ORR: 10 (90.9%) - CR: 7 (63.6)	3 (27.3%)			
Jacobson et al. [33], 2020 (ZUMA-6 trial)	B cell-NHL	Axi-cel + Atezolizumab	28	ORR: 21 (75.0%) - CR: 13 (46.4%)		50% #	71% #	62% #
Ramakrishnan et al. [34], 2020 (ALEXANDER trial)	B cell-NHL	AUTO3 + Pembrolizumab	33 (29 *)	ORR: 20 (69.0%) - CR: 15 (51.7%)				
Chong et al. [35], 2022	B cell-NHL	Tisa-cel + Pembrolizumab	12	ORR: 3 (25.0%) - CR: 1 (8.3%) - PR: 2 (16.7%) PD: 8 (66.7%) SD: 1 (8.3%)	10 (83.3%)	2.8 (0.4–35.2)		
Hirayama et al. [36], 2022	B cell-NHL	Anti-CD19+ Durvalumab	29 (26 *)	ORR: 9 (34.6%) - RC: 7 (26.9%)				
Jaeger et al. [37], 2023 (PORTIA trial)	B cell-NHL	Tisa-cel + Pembrolizumab	12	ORR: 6 (50.0%) - CR: 4 (33.3%) - PR: 2 (16.7%) PD: 6 (50.0%)	0%			

* Evaluable for response; # Estimated at 6 months. Abbreviations: B-ALL, B-cell acute lymphoblastic leukemia; B cell-NHL, B-cell no Hodgkin lymphoma: ORR, Objective response rate; SD, Stable disease CR, Rate of complete response; PR, Rate of partial response; DOR, duration of response; PFS, progression-free survival; OS, Overall survival.

Jacobson et al. [33] presented at the American Association for Cancer Research virtual annual meeting 2020 the primary analysis of phase I and II ZUMA-6 trial in patients with R/R DLBCL who received axi-cel and atezolizumab 1200 mg every 21 days (a total of four doses) with three cohorts in phase I and for phase II it was decided to use the dosing schedule of Cohort 3. Eighteen patients received the full four-dose regimen, and twenty-eight received at least one dose. Most patients (86%) received ≥ 2 prior treatments. With a median follow-up of 10.2 months, the best ORR was 75%, and 46% of patients remained in response at the time of analysis. Median peak CAR-T cell levels and median CAR-T cell expansion measured by the area under the curve (AUC) in the first 28 days were similar between the ZUMA-6 trial [33] and ZUMA-1 trial, which studied axi-cel alone [15,21].

Ramakrishnan et al. [34] analysed another strategy to target tumour antigens in addition to disrupting PD-1/PD-L1 interaction using bicistronic CAR-T (or dual CAR-T) cells targeting the two antigens CD19 and CD22 (AUTO3), followed by consolidation with anti-PD-1 (three doses of pembrolizumab 200 mg) in 33 patients with R/R DLBCL with a median number of previous lines of treatment of three (1–10). Efficacy was assessed in 29 patients. A total of 29 patients were evaluated for efficacy, with an ORR of 69%. Fourteen out of fifteen CRs did not experience relapse, with a median follow-up of 3 months (1–24 months). Among the 15 evaluable patients treated at a dose > 50 × 10e6 with D-1 pembrolizumab, the ORR was 73%, and no relapse was observed among CR patients. Results about relevant biomarkers have not yet been reported.

Chong et al. [35] published the results of their phase I/II trial in patients with B-NHL R/R to CAR-T therapy with tisagenlecleucel in 2022. The study enrolled 12 patients with B-NHL who had undergone a median of four prior lines of treatment (ranging from three to eight). As part of the treatment approach, patients received pembrolizumab at 200 mg/day every 3 weeks until DP or unacceptable toxicity. The median time from CAR-T cell infusion to the first pembrolizumab dose was 3.3 months, with a range of 0.4–42.8 months. Among the 12 assessable patients, the best ORR after pembrolizumab was 25% (3/12). These responses occurred within 1 to 3 months of the beginning of pembrolizumab. The patient with FL did not respond to pembrolizumab treatment. Tisagenleculeucel re-expansion was observed in 10 patients (responders showed more than one re-expansion peak during pembrolizumab, while non-responders either had just one peak or none at all).

Hirayama et al. [36] launched a phase IB trial in patients with R/R CD19-positive NHL-B to evaluate JCAR014, an autologous anti-CD19 CAR-T therapy, in combination with durvalumab (with dose escalation 21–28 days after or 1 day before JCAR014 infusion). The most recent available results presented at the American Society of Haematology Annual Meeting in 2022 included 29 patients (all patients received 2 × 10e6 JCAR014 cells/kg, except for the first 2 pts treated on the study who received 7 × 10e5 cells/kg). Twenty-six were evaluable for response. The ORR and the CR rate at 3 months reported were retrospectively compared to those found in patients who received only JCAR014 dose (2 × 10e6 cells/kg) without durvalumab from their previous phase I/II clinical trial. The analysis did not reveal any significant differences in ORR and CR rates between the two groups of patients, but there was a trend toward lower response for the combined therapy. They did not observe significant differences either in peak CAR-T cell expansion, in AUC from day 0 to 28, or in the CAR-T cell counts on day 28. Also, they observed that the initiation time of durvalumab therapy is a key variable that may affect outcomes and patients treated with JCAR014 alone.

Jaeger et al. [37] conducted the PORTIA trial in patients with R/R DLBCL refractory who were refractory to two or more lines of treatment. The regimen treatment consisted of tisagenlecleucel on day 1 and pembrolizumab 200 mg every 21 days for up to six cycles. The main objectives of the study were to analyse the proportion of patients receiving pembrolizumab according to protocol, the incidence of DLT in the dose escalation phase, the duration of response, and ORR. Three cohorts initiated pembrolizumab on days 15 (n = 4), 8 (n = 4), or –1 (n = 4). Median follow-up from tisagenlecleucel infusion was 230 days, and 6/12 responded to the treatment. The D–1 cohort had the highest ORR (75%; 95% CI, 19.41–99.37); three patients achieved sustained CR. Two (50%) patients from the D15 cohort achieved PR (95% CI, 6.76–93.24), and one (25%) from the D8 cohort achieved CR (95% CI, 0.63–80.59). Markedly, pembrolizumab did not result in a secondary expansion of tisagenlecleucel, regardless of the number of doses.

### 3.5. Safety Results

The main adverse events related to CAR-T treatments are detailed in Table 3. Cytokine release syndrome (CRS) occurred in 7/9 studies. Grade 3 CRS was reported by Jaeger et al. [37] (8%), Hirayama et al. [36] C (7%), and Jacobson et al. [33] (4% of patients). The remaining studies reported grade 1 or 2 CRS in a higher percentage of treated patients.

Neurotoxicity (any grade) was reported by 4/9 studies. Grade 3 neurotoxicity was observed in 29% of patients treated with axi-cel + atezolizumab and 7% of patients treated with anti-CD19 and durvalumab [36].

Five studies showed cytopenias related to the treatment. Three of them [28,34,35] detected grade 3 or higher cytopenias. Thrombocytopenia and grade 2 anaemia were reported in 27% and 18% of patients treated with anti-CD19 + nivolumab [30] and grade 3 in 58% and 47% of patients treated with anti-CD19/22 + pembrolizumab [34], both chemotherapy associated.

Toxicities were manageable and reversible in all studies. No AEs, autoimmune adverse events, or treatment-attributable deaths were reported in eight studies. Only Hirayama et al. [36] reported one patient who experienced grade 4 SLC and extensive bone marrow infiltration and died early.

## 4. Discussion

The management of patients with relapsed or refractory haematological malignancies, particularly those with R/R B-NHL or acute B-ALL after several lines of treatment, remains a challenge for clinicians. This is mainly due to the high mortality rates associated with these pathologies, the limited therapeutic options available, and the great uncertainty regarding their efficacy and safety [11,14]. In this scenario, CAR-T therapies combined with ICI have been postulated as a golden future treatment opportunity.

This paper evaluates the available evidence on the efficacy and safety of combined or sequential use of programmed cell death-1 (PD-1 or PD-L1) receptor inhibitors and CAR-T therapies in managing NH relapsed or refractory to other treatments. We include nine studies reporting results about this association published up to date. The main pathologies in which it is used are B-ALL and B-NHL, and the most studied combination is the association of tisagenlecleucel with pembrolizumab [29,35,37]. However, the available evidence on the efficacy and safety is still limited. Additionally, most clinical trials in this context are still ongoing. These trials are designed as open-label, single-arm phase I/II trials, and, in some cases, they are conducted at a single centre. The primary objective of these trials is to assess the safety of the combination therapy.

Based on the studies published so far, the therapy appears to be safe and well tolerated. The AEs observed in these trials and real-life studies for the various CAR-T therapies used as monotherapy. Importantly, most of these AEs were not serious. Due to the variability between studies and the lack of data on some of them, it is difficult to know the influence that the time interval between CAR-T cell and ICI therapies could have on the incidence of adverse reactions.

Regarding preliminary efficacy results, there are heterogeneous findings across studies depending on the pathology.

In B-ALL patients, the combination of anti-CD19 CAR therapy with pembrolizumab or nivolumab was evaluated for the treatment of no-response or relapse after CAR-T cell therapy. In that context, it appears to hold promise, as indicated by studies conducted by Maude et al. [29] and Li et al. [28], in which, with the addition of ICI, a CAR was detected with re-expansion in all cases and achieving very acceptable response rates, especially considering that the patients included in the trials were heavily pre-treated. Li et al. [28] observed promising responses were therefore observed specifically in those with early B-cell recovery and bulky extramedullary disease. However, PD-1 inhibition had a partial but not long-lasting effect in the four B-ALL patients with a poor initial marrow response to CAR-T cell therapy. Both the ORR and CR rates reported by Maude et al. [29] (50% and 25%, respectively) were slightly higher than those reported by Li et al. [28] (42.9% and 14.3%). Although the sample size of both studies is low (4 vs. 14), the findings of both studies show positive results for the combination of these two therapies.

However, studies conducted in patients with B-NHL focused on analysing the effect of the administration of ICI as an adjuvant treatment to augment the efficacy of CAR-T cells, and there is considerable heterogeneity comparing their findings and reported efficacy results. The high ORRs obtained with liso-cel + durvalumab by Siddiqi et al. [31], anti-CD19 + nivolumab in the study performed by Cao et al. [38], with axi-cel + atezolizumab in the study of Jacobson et al. [33], and with anti-CD19/22 + pembrolizumab in the study conducted by Ramakrishnan et al. [34] (90.9%, N = 11; 81.8%, N = 11; and 75%, N = 28 and 69%, N = 33, respectively) contrast with those reported for anti-CD19 + pembrolizumab by Chong et al. [35] and for anti-CD19 + durvalumab by Hirayama et al. [36] (25% and 34.6%), while Jaeger et al. [37] reported an ORR of 50% for tisa-cel + pembrolizumab. Similarly, CR rates vary from 63.6%, 45.5%, 46.4%, and 51.7% observed by Siddiqi et al. [31], Cao et al. [30], Jacobson et al. [33], and Ramakrishnan et al. [34], respectively, to 8%, 27%, and 17% reported by other authors (Table 2).

Three studies provide PFS data, ranging from 2.8 months (Chong et al. [35]) to 6 months (Cao et al. [30] and Jacobson et al. [33]). Only the study by Jacobson et al. [33] provides OS data with an estimated at 6 months OS of 71%.

Concerning CAR expansion, unlike in studies involving patients with B-ALL, studies in B-NHL do not always show CAR re-expansion after ICI administration. In the study by Chong et al. [35], 83.3% CAR re-expansion was found after pembrolizumab administration. In addition, they observed a possible relationship between the achieved response and the number of episodes of CAR-T re-expansion, and their analyses indicate increased CAR-T cell activation and proliferation with decreased markers of exhaustion after pembrolizumab in patients responding to pembrolizumab after CAR-T cell therapy. However, re-expansion peaks observed in patients with clinical benefit did not temporally correlate with doses of pembrolizumab. Siddiqi et al. [31] observed reassembling of CAR-T cells after durvalumab administration in only 27.3% of patients, while in the work of Jaeger et al. [37] and Jacobson et al. [33], the overall CAR exposure was consistent with that observed in the JULIET [3] and ZUMA-1 [15,21] trials, respectively, and there was no secondary CAR expansion after ICI administration. Hirayama et al. [36] also did not find significant differences for CAR-T cells +/− durvalumab cohorts in this respect, and Cao et al. [30] also stated that the number of CD19 CAR-T cells in their patients did not seem to be higher than the number measured in the patients in their centre who received CD19 CAR-T cell infusion alone, despite the high response rate reported. However, they recognised that patients who received the combination had more adverse prognostic factors. What does show consistency across studies is that CAR re-expansion as a result of ICI use is mainly limited to responders.

CAR-T cell therapies combined with ICI have been postulated as a golden opportunity for treatment, according to preliminary data, in those patients with a poor response to CAR [19,24,28]. The problem with the expression of immunosuppressive substances is that CAR-T cells become exhausted and decrease their quality or their number. To solve this problem, the development of sophisticated therapies enhancing therapeutic efficacy is underway. Despite the positive results, considering the response rates shown in some of the papers, it appears that the addition of ICI to anti-CD19 CAR-T cell therapy is not superior to CAR-T cell monotherapy. Even with the limitations of indirect comparisons across studies (especially due to different patient characteristics) [30,33,37], it appears that the addition of ICI to anti-CD19 CAR-T cell therapy is not superior to CAR-T cell monotherapy and would not provide an improvement or added benefit to the main efficacy and CAR exposure/expansion outcomes shown in pivotal trials or previous studies of these therapies.

Prior works analysed the role of immunotherapy, particularly PD-1 and PDL-1 blockade and CAR-T cell therapies, in the management of different haematological malignancies [24,25,38,39]. These authors postulated the combination of these therapies as a treatment opportunity in certain scenarios due to the high potential of the preclinical data shown in studies. However, well-designed clinical trials with many patients are needed to evaluate their results based on efficacy and safety. They emphasised that it was a combination therapy, with no serious toxicities in addition to those already known, although immuno-related toxicities would need to be closely monitored. However, they stated that there was still a way to achieve maximum optimisation of the efficacy of CAR-T cell therapy in terms of the drugs of choice, doses, times, and sequences of administration, as well as the profile of patients who were candidates to receive them. In addition, evidence is being generated on the potential role of ICIs as salvage therapy in patients with NHL who progress after receiving CAR-T cell therapy [40].

In contrast to other reviews that only refer to ongoing clinical trials in this setting [25], our work focused on the selection of clinical trials and studies evaluating haematological malignancies that provide updated efficacy and safety data, even though some studies only have preliminary results. In any case, we can state that the reporting results showed that more studies with a larger number of patients and longer follow-up periods are needed to obtain mature efficacy and safety data, allowing conclusions to be drawn with greater clinical evidence.

The main limitation of the study was the difficulty of synthesizing the reporting results due to the high variability between the studies in terms of design, type of population, pharmacotherapeutic combination, follow-up period, and efficacy variables, among others. Consequently, it constitutes an important obstacle to obtaining clear conclusions on efficacy and safety. On the other hand, the available evidence for ICIs together with CAR-T cell therapy (concomitant or sequential) is, to date, low. The data are still quite preliminary, and the best-designed clinical trials are ongoing, so their findings are only partially incorporated in this paper. The number of patients for whom published data exist are small cohorts from clinical trials and, therefore, may be insufficient to extrapolate the findings to clinical practice.

Furthermore, due to the immaturity of the data, the median follow-up times of the patients are rather short, so there is great uncertainty about the long-term effect of the combination therapy of ICI with CAR-T cells, especially on patient survival, as the available results are very limited.

## 5. Conclusions

Despite the existence of a large number of studies focused on the synergistic effect of the combination of ICI with CAR-T therapy, the current clinical evidence on toxicity and efficacy is limited because most of the studies have very preliminary data in small population size, and others are clinical trials that are in very early stages and still ongoing. The main pathologies in which this combination therapy has been used are B-ALL and B-NHL. The most studied therapeutic combination is the combination of tisagenlecleucel with pembrolizumab.

For the moment, the efficacy of combined or sequential treatment of PD1/PDL1 inhibitors and CAR-T therapies for managing haematological malignancies shows heterogeneous efficacy results. Evidence suggests that combining tisagenlecleucel with pembrolizumab could be a promising option in B-ALL with modest data. In B-NHL, although with hopeful responses, the combination does not appear better than CAR-T cell monotherapy based on the findings observed. In any case, due to the limitations of studies, it is difficult to know if the association would provide a real added benefit, so further prospective trials should be needed. The combination of CAR-T cell therapy with ICIs was well tolerated with manageable toxicities, whose major adverse events resembled those of CAR-T cell monotherapy.

## Figures and Tables

**Table 3:** Toxicity results: treatment-related adverse events (TRAEs).

Author, Year	N	TRAEs	Any Grade N (%)	Grade 3–4 N (%)
Li et al. [28], 2017	14	Cytopenia	4 (28.6%)	4 (28.6%)
		CRS	3 (21.4%)	-
		Fever	3 (21.4%)	-
		Acute pancreatitis	1 (7.1%)	-
		Hypothyroidism	1 (7.1%)	-
		Arthralgias	1 (7.1%)	-
		Urticaria	1 (7.1%)	-
Maude et al. [29], 2017	4	Fever (without CRS)	2 (50.0%)	-
Cao et al. [30], 2019	11	CRS (82)	9 (81.8%)	-
		Neurotoxicity (9)	1 (9.1%)	-
Siddiqi et al. [31], 2019 (PLATFORM study)	11	Fever	NR	-
		CRS		-
		Fatigue		-
		Cytopenia		-
		Haemolytic anaemia		-
		Rash		-
		Neurotoxicity		-
Jacobson et al. [33], 2020 (ZUMA-6 trial)	28	Neurotoxicity	8 (28.6%)	8 (28.6%)
		CRS	1 (3.6%)	1 (3.6%)
Ramakrishnan et al. [34], 2020 (ALEXANDER trial)	33	Neutropenia	24 (72.7%)	24 (72.7%)
		Thrombocytopenia	21 (63.6%)	16 (48.5%)
		Anaemia	20 (60.6%)	16 (48.5%)
		CRS	11 (33.3%)	-
		Pyrexia	10 (30.3%)	-
		Constipation	9 (27.3%)	-
		Fatigue	8 (24.2%)	
Chong et al. [35], 2022	12	Neutropenia	4 (33.3%)	3 (25.0%)
		Fever (without CRS)	3 (25.0%)	-
Hirayama et al. [36], 2022	29	CRS	12 (41.4%)	2 (6.9%)
		Neutropenia	6 (20.7%)	-
		Neurotoxicity	5 (17.2%)	-
		Hypogammaglobulinemia	5 (17.2%)	-
Jaeger et al. [37], 2023 (PORTIA trial)	12	CRS	7 (58.3%)	1 (8.3%)
		Neutropenia	2 (16.7%)	2 (16.7%)
		Anaemia	2 (16.7%)	1 (8.3%)
		Lymphopenia	2 (16.7%)	1 (8.3%)

Abbreviations: CRS, cytokine release syndrome; NR: no data reported.

## Data Availability

Not applicable.
